# Updates on Antibiotic Regimens in Acute Cholecystitis

**DOI:** 10.3390/medicina60071040

**Published:** 2024-06-25

**Authors:** Valeria Fico, Antonio La Greca, Giuseppe Tropeano, Marta Di Grezia, Maria Michela Chiarello, Giuseppe Brisinda, Gabriele Sganga

**Affiliations:** 1 Emergency Surgery and Trauma Center, Department of Abdominal and Endocrine Metabolic Medical and Surgical Sciences, Istituto di Ricerca e Cura a Carattere Scientifico, Fondazione Policlinico Universitario Agostino Gemelli, 00168 Rome, Italy; valeria.fico@policlinicogemelli.it (V.F.); antonio.lagreca@policlinicogemelli.it (A.L.G.); giuseppe.tropeano@guest.policlinicogemelli.it (G.T.); marta.digrezia@policlinicogemelli.it (M.D.G.); gabriele.sganga@policlinicogemelli.it (G.S.); 2 Catholic School of Medicine “Agostino Gemelli”, 00168 Rome, Italy; 3 General Surgery Operative Unit, Department of Surgery, Azienda Sanitaria Provinciale Cosenza, 87100 Cosenza, Italy; michela.chiarello@aspcs.it

**Keywords:** acute cholecystitis, antibiotic therapy, antimicrobial therapy, antibiotic resistance, biliary infections, microbiology

## Abstract

Acute cholecystitis is one of the most common surgical diseases, which may progress from mild to severe cases. When combined with bacteremia, the mortality rate of acute cholecystitis reaches up to 10–20%. The standard of care in patients with acute cholecystitis is early laparoscopic cholecystectomy. Percutaneous cholecystostomy or endoscopic procedures are alternative treatments in selective cases. Nevertheless, antibiotic therapy plays a key role in preventing surgical complications and limiting the systemic inflammatory response, especially in patients with moderate to severe cholecystitis. Patients with acute cholecystitis have a bile bacterial colonization rate of 35–60%. The most frequently isolated microorganisms are *Escherichia coli*, *Klebsiella* spp., *Streptococcus* spp., *Enterococcus* spp., and *Clostridium* spp. Early empirical antimicrobial therapy along with source control of infection is the cornerstone for a successful treatment. In these cases, the choice of antibiotic must be made considering some factors (e.g., the severity of the clinical manifestations, the onset of the infection if acquired in hospital or in the community, the penetration of the drug into the bile, and any drug resistance). Furthermore, therapy must be modified based on bile cultures in cases of severe cholecystitis. Antibiotic stewardship is the key to the correct management of bile-related infections. It is necessary to be aware of the appropriate therapeutic scheme and its precise duration. The appropriate use of antibiotic agents is crucial and should be integrated into good clinical practice and standards of care.

## 1. Introduction

Acute cholecystitis (AC) is worldwide one of the most frequent causes of hospitalization, and it is the second most frequent cause of surgical emergency admission in the Western world [1,2].

AC is defined as a clinical syndrome distinguished by right upper quadrant pain, fever, and leukocytosis associated with gallbladder inflammation. In most cases, AC is the most common complication of biliary lithiasis and typically develops in patients with a history of symptomatic gallstones. Worldwide, the estimated prevalence of gallstones in the general population is 10–15%, and AC represents the first clinical presentation in 10–15% of cases [3,4]. In one systematic review, AC developed in 6 to 11 percent of patients with asymptomatic and symptomatic gallstones over a median follow-up of 7 to 11 years [5]. These data become even more relevant considering that, according to the 2017 United Nations report, the population over 60 will reach 35% in Europe in 2050 [6] and that the prevalence of gallstone disease increases with age [7].

Early identification of symptoms allows treatment to start as soon as possible, reducing the risk of developing complications like localized perforation or peritonitis (in 10% and 1% of non-treated patients, respectively) [8]. The bile, sterile in the initial stages of AC, ends up becoming infected [9]. The mortality rate of severe biliary infections ranges from 1% to 6%, reaching 10–20% when combined with bacteremia. This is crucial, especially in the elderly, in which bacteremia following the biliary tract infection is the second most frequent cause of sepsis [10,11].

Approximately 5% to 10% of cases of cholecystitis may not be related to the presence of gallstones [12]. It is very often diagnosed in immunosuppressed patients, due to the greater susceptibility of these patients to certain opportunistic infections such as *Cytomegalovirus* and *Cryptosporidium*, which can proliferate in the bile [13]. Acute acalculous cholecystitis (AAC) can be distinguished into two forms: primary AAC, associated with a pre-existing severe disease, and secondary AAC, which may complicate systemic bacterial, viral, or fungal infections [14,15]. Other risk factors for the development of AAC are total parenteral nutrition, intensive care unit hospitalization, major surgeries, heart attack, stroke, sepsis, severe burns, and extensive trauma [16]. Furthermore, patient carriers of *Giardia lamblia*, *Salmonella typhi,* and *Helicobacter pylori* also have a greater risk of developing AAC [17]. AAC is a life-threatening disorder, with a high incidence of perforation and gallbladder gangrene (10–15% and 50%, respectively). Even in the case of AAC, bacterial infection occurs secondarily [14].

The standard of care in patients with both AC and AAC is early laparoscopic cholecystectomy [18,19,20,21,22]. In fact, when treated only with antibiotics, patients show recurrence of symptoms in 2.5–22% of cases [23]. Nevertheless, the goal for the treatment of moderate to severe AC is source control. Antimicrobial therapy plays a pivotal role in preventing surgical complications and limiting the systemic inflammatory response.

The aim of this review is to focus on the latest scientific evidence on antibiotic therapy in AC, considering the rising problem of antimicrobial resistance.

## 2. Pathogenesis and Diagnosis

AC is usually caused by cystic duct obstruction and the irritating effects of stones on the gallbladder wall. Cystic duct obstruction alone, in fact, is not enough to explain the pathogenesis of gallbladder inflammation [24,25].

In experimental models, lysolecithin, a product derived from lecithin and produced during gallbladder wall micro trauma, is used to induce gallbladder inflammation. This process is in vivo activated by phospholipase A, which is normally present within the mucosa and released when its integrity is disrupted (for example, due to the mechanical effects of stones). This molecule is, in fact, detectable in gallbladder bile in patients with AC [26]. The propagation of the inflammation is sustained by inflammatory mediators, i.e., prostaglandins, which have a role in the contraction of the gallbladder and in the absorption of fluids [27,28]. This hypothesis is supported by the relief of symptoms during biliary colic when treated with a prostaglandin inhibitor [29,30]. Besides the inflammatory process, in some cases, the situation is worsened by bile infection [31]. Histologic changes in the gallbladder in AC can range from mild edema and acute inflammation to necrosis and gangrene. 

AC should be suspected in patients who complain of pain and tenderness localized to the right hypochondrium or epigastrium, with or without guarding, and a systemic inflammatory response with fever, leukocytosis, and raised C-reactive protein [32]. A positive Murphy’s sign (pain in the right upper quadrant during inspiration) supports the diagnosis. However, clinical history, clinical manifestations, and laboratory tests are not sufficient to establish a diagnosis. The demonstration of gallbladder wall thickening or edema or ultrasound Murphy’s sign is required for diagnosis.

With the aim of clearly defining the diagnostic criteria of AC, guidelines were drawn up in 2007 and subsequently revised in 2018, known as the Tokyo Guidelines [33]. We can distinguish three grades of cholecystitis as follows:

- Grade 1: mild gallbladder inflammation; no organ dysfunction;
- Grade 2: moderate AC with any of the following but no organ dysfunction: WBC > 18 × 10^9^/L, palpable tender mass in the upper right quadrant, duration of complaints exceeding 72 h, and marked local inflammation (biliary peritonitis, abscesses, and gangrenous cholecystitis);
- Grade 3: severe AC with at least one organ dysfunction: cardiovascular dysfunction, neurological dysfunction, respiratory dysfunction, renal dysfunction, and hepatic dysfunction (INR > 1.5).

The diagnosis of AAC can be challenging, especially in critically ill patients and often the precise etiology remains unknown [34].

## 3. Treatment

The definitive treatment of AC is represented by cholecystectomy or, in the case of high-risk patients, by percutaneous cholecystostomy performed by interventional radiologists or gallbladder drainage through endoscopic procedures [35,36,37]. Definitive treatment of AC (operative vs conservative) depends on the time since the onset of symptoms, the patient’s comorbidities, and clinical presentation [38,39]. When a patient suffering from cholelithiasis develops AC, definitive treatment is recommended because of the high risk of the recurrence of symptoms or onset of complications [40]. In a trial of the National Cooperative Gallstone Study on non-surgical treatment of symptomatic cholelithiasis, patients presented a recurrence of symptoms in approximately 70% of cases in the two years following the first clinic manifestation [41]. In a study carried out on 25,397 patients hospitalized following a first episode of AC, 10,304 did not undergo cholecystectomy. Among these patients, 24% recurred with symptoms related to the presence of gallbladder lithiasis in the following 3 years; most patients (88%) presented a recurrence in the first year after the first manifestation [42]. For AAC, the treatment of choice is cholecystectomy, even if percutaneous cholecystostomy represents a valid alternative in patients with organ failure or hemodynamic instability [43]. The remaining therapy consists of supportive medical therapy (NSAIDs, antiemetics, and intravenous hydration) and antibiotic therapy if necessary.

AC is always characterized by an inflammation process, but infection is not always present. Gallbladder inflammation is caused by bile stasis, which determines parietal ischemia and irritation; in the first phase of this process, especially in community-acquired infections, bile is often sterile [44]. The biliary tract is physiologically sterile since bile contains products that cause bacterial cell wall breakdown. These products are lipopolysaccharides and lipoteichoic acid. Furthermore, biliary tree epithelial cells produce a mucus layer by which immunoglobulin can easily reach the bile duct [45,46]. These mechanisms help to maintain low bacterial contamination in physiological conditions. On the other hand, the stasis can lead to a bacterial translocation or to a superinfection of the bile [47,48]. It has been demonstrated that the presence of bacteria in bile cultures varies from 41 to 63%, especially in the initial stages of the disease [49]. It is controversial how long this initial phase of inflammation lasts. Järvinen, in a series of 515 patients, demonstrated positive intra-operative bile cultures in 63% of patients operated on within 24 h of the onset of AC, while in the group receiving delayed surgery greater than or equal to 11 days later, the percentage was reduced to 31% [50]. Several studies have examined the risk factors for the infection of the gallbladder bile. Thompson et al. report an association of bile infection with fever > 37.5 °C, a total bilirubin level >8.5 mg/dL, and a WBC > 14 × 10^9^/L [51].

## 4. Microbiology

In community-acquired AC there is almost always a mixed infection [49,52]. The pathogen spectrum derives from the flora of the gastrointestinal tract. Key pathogens are *Escherichia coli*, *Bacteroides fragilis,* and Enterococci. Resistant species need only be considered in patients treated with antibiotics on an outpatient basis [53,54,55]. Other specific risk factors are as follows:

1. Antibiotic treatment of other illnesses (such as infected diabetic foot, pneumonia);
2. Relocation from countries/regions with a high prevalence of resistant pathogens;
3. Patients coming from countries with high multidrug-resistant (MDR) organism prevalence;
4. Known MDR organism colonization of the gastrointestinal tract;
5. Immunosuppression;
6. Patients coming from healthcare residences;
7. Prolonged hospital/intensive care stay.

A bile bacterial colonization rate is reported among 35–60% of patients with AC [49]. Few data have been published on bacteriological analysis of bile in patients affected by AC [44]. Most biliary infections (80%) are polymicrobial, and severe cases are often associated with bacteremia [14]. Although there are not much data on the microbiology of AC, bile culture is strongly recommended in all cases with the exception of grade 1 according to Tokyo guidelines [33]. Enteric microorganisms have been isolated in 70% of cultures. According to Asai et al., the most frequently isolated microorganisms are *Escherichia coli* (39.4%), *Klebsiella* spp. (35.1%), *Streptococcus* spp. (18.1%), *Enterococcus* spp. (17.0%), *Enterobacter* spp. (10.6%), *Pseudomonas aeruginosa* (4.3%), and anaerobes (17.0%), including *Clostridium* spp. (13.8%) and *Bacteroides* spp. (3.2%) [56]. Gomi et al., in a large multicenter study, reported *Escherichia coli* as the most frequently isolated microorganism [57]. Only 20% of cases are sustained by Gram-positive cocci; among these, *Enterococcus* spp. has been detected in up to 34% of cases [48]. No statistically significant differences are reported either in terms of isolated pathogens or in the incidence of multi-resistant germs in relation to the severity of the clinical presentation [48]. Analyzing the intestinal microbiota of AC patients, intestinal dysbiosis was observed. Compared to healthy controls, there is a predominance of *Akkermansia*, *Enterobacteriaceae*, *Escherichia coli,* and *Shigella*. On the other hand, healthy patients have a prevalence of other microorganisms such as *Clostridiales*, *Coprococcus*, *Coprobacillacea*, *Paraprevotella*, *Turicibacter*, *Faecalibacterium*, and *Ruminococcus* [58].

The same changes have been detected in the biliary flora; the hypotheses for this contamination are mainly represented by the ascending biliary route and the portal hematogenous route [59]. Another factor linked to intestinal microbiota alterations is the development of antibiotic resistance. This phenomenon appears to be partly linked to the extensive use of oral fluoroquinolones for genitourinary tract infections, pneumonia, and soft tissue infections, which have progressively changed the intestinal microbiota in favor of Gram-negative enterobacteria [60]. As confirmed by Liu et al., enterobacteria are responsible for several infectious diseases such as urogenital tract infections and necrotizing enterocolitis [59]. They have demonstrated that the inoculation of Enterobacteriaceae isolated from patients with AC (1 × 10^8^ CFU of *Escherichia coli*, strain O124:K72, pathogenic type EIEC), in guinea pigs by gastric or rectal methods, was alone capable of morphological changes in the gallbladder, stone formation, and bile composition variations. Otherwise, non-inoculated pigs presented clear and limpid bile, anatomically normal gallbladder, and absence of stones at the anatomical pathological analysis performed 1 month after the inoculation [59].

Different studies report a high incidence of antibiotic resistance among bacteria isolated in bile cultures. A worldwide multicenter study by Coccolini et al. reports a 16.7% increase in extended-spectrum beta-lactamase (ESBL)-producing microorganisms among isolated *Escherichia coli* [61]. The growing incidence of multidrug-resistant (MDR) organisms may lead to an increase in treatment failures because some antibiotics, such as amoxicillin–clavulanate and some cephalosporins and fluoroquinolones, can no longer be used empirically in many regions of the world [9,62]. Goo et al. highlighted that 3.5% of isolated germs were carbapenemase producers [63]. The antibiotic resistance profile varies from region to region. Therefore, it is important to monitor and update the local profile of antibiotic resistance, which is critically important for the correct choice of empirical antibiotic therapy. Another important consideration is the difference between community-acquired infections (CAIs), healthcare-associated infections (HCAIs), and hospital-acquired infections (HAIs). An increased isolation of *Enterococcus* and *Pseudomonas* was detected in cases of HAIs, as well as *Escherichia coli* and *Klebsiella* resistant to fluoroquinolones (22%) [64]. Moreover, it should be noted that the bile culture of patients with biliary stents is different, with a larger presence of Enterococci and non-fermenting Gram-negative bacteria, particularly *Enterococcus faecium* and *Pseudomonas aeruginosa* [65]. *E. faecium* represents a cause for concern due to its intrinsic resistance to common antibiotics. This germ has been isolated in up to 12% of patients with AC, 17% with AAC, and 45% with cholangitis [66]. The percentage of ESBL-producing microorganisms is also increasing [64].

## 5. Antimicrobial Therapy

Early empirical antimicrobial therapy along with source control of infection is the cornerstone for a successful treatment which, if inadequate, represents an independent predictor factor of mortality [67,68]. The Surgical Infection Society Guidelines recommend a maximum of four days of antibiotic therapy in patients with severe AC undergoing surgery; on the other hand, they recommend against the use of post-operative antibiotics in patients with mild or moderate AC [69]. The severity grading of AC according to Tokyo guidelines can be used to select antibiotics and determine the best approach for septic source control [14]. In the management of critically ill patients with AC, the choice of the antimicrobial regimen may be challenging. It is recommended to start broad-spectrum intravenous antibiotics within the first hour from the onset of septic shock symptoms, and within six hours in the other cases [70,71,72]. Other crucial diagnostic and therapeutic measures include blood cultures, measurement of serum lactate, resuscitation with intravenous crystalloids, and, in the case of life-threatening hypotension, vasopressor drugs. According to the World Society of Emergency Surgery guidelines, empirical antibiotic treatment should include molecules active on the most frequently isolated aerobic and anaerobic Gram-negative bacteria (*Escherichia coli*, *Klebsiella,* and *Bacteroides*) [73]. The potential pathogenicity of Gram-positive enterococci is not clear and targeted antibiotic therapy should not be prescribed for community-acquired infections; it should be administered, instead, for immunosuppressed or transplanted patients. Furthermore, ESBL-producing microorganisms are frequently isolated in patients with community-acquired biliary infections previously treated with antibiotics [74]. Pharmacological administration should be chosen according to the most frequently isolated microorganisms, also considering the local trends of antibiotic resistance and drug availability. Moreover, the drug choice will depend on whether the infection is community-acquired or hospital-acquired, its severity, and the risk of the presence of MDR microorganisms [14].

In critically ill patients, the choice of antimicrobial regimen may be complex, especially for healthcare-associated infections caused by resistant bacteria. For these patients, the early identification of the responsible microorganism, by bile cultures, represents the crucial step in the management of AC [74,75]. Furthermore, the chosen molecule should have a good biliary distribution and one of the principles of correct prescription is to choose the least costly effective antibiotic. Overall, several classes of antibiotics have good biliary penetration profiles: piperacillin–tazobactam, tigecycline, amoxicillin–clavulanate, ciprofloxacin, and ampicillin–sulbactam cefotaxime and ceftazidime have good bile penetration. On the other hand, other antibiotics such as meropenem may have impaired pharmacokinetics due to biliary obstruction [76], and vancomycin and aminoglycoside, have low bile penetration [77]. It must be considered that in patients with biliary obstruction, the penetration of antibiotics into the bile may be reduced [78].

According to the above, penicillins are frequently used in biliary infections. Biliary concentrations of ampicillin are at least three times the corresponding serum concentrations [79], piperacillin is significantly excreted in the bile [80], and amoxicillin concentration falls below the susceptibility breakpoints of common bile tract infection pathogens [81]. Amoxicillin–clavulanic acid, aminoglycosides, piperacillin–tazobactam, and carbapenems are also active against the usual Gram-negative bacteria. Ceftriaxone demonstrated higher levels of biliary excretion than other third-generation cephalosporins [82]. Metronidazole appears rapidly in the bile. For empiric anaerobic coverage of moderate-to-severe cholangitis or severe cholecystitis, metronidazole should be used in combination with a fluoroquinolone, a third-generation or fourth-generation cephalosporin, or aztreonam [83]. Fluoroquinolones have excellent bioavailability in the bile tract where the concentration of ciprofloxacin is 28 to 45 times higher than the plasma concentration. Fluoroquinolones are not recommended in some European countries due to their loss of efficacy against these bacteria (between 30 and 55% in the case of resistant *E. coli*) and their poor efficacy against streptococci and enterococci [84]. The combination of ciprofloxacin with metronidazole may be an alternative to amoxicillin–clavulanic acid in patients with mild or moderate AC and no risk factors for resistance [8]. The glycylcyclines, such as tigecycline, have a broad spectrum of activity and good availability in bile and in the gallbladder wall [85]. Since most cephalosporins, penicillins, aminoglycosides, and carbapenems are excreted by the kidneys, their dose should be reduced in patients with impaired renal function [86]. Concerning Gram-positive strains, they maintain high sensitivity to beta-lactams, except for *E. faecium*, which is sensitive to glycopeptides, daptomycin, and linezolid. Piperacillin–tazobactam, carbapenems, or ceftriaxone reach high bile concentrations but may select vancomycin-resistant enterococci [87].

In cases of community-acquired bacteremia and in immunocompromised patients, transplant recipients or those previously treated with antibiotics, ESBL-producing Enterobacteriaceae or *Pseudomonas* spp. may be present and initial empiric treatment with piperacillin–tazobactam or a carbapenem should be performed [64]. Critically ill patients with AAC are at elevated risk of infection by *Candida* spp, so fluconazole or echinocandin should be added to antibiotic therapy, using echinocandin only in patients recently treated with fluconazole or in those with septic shock. Table 1 summarizes the most appropriate antibiotic strategies in the case of community-acquired infection.

Table 2 summarizes the most appropriate antibiotic strategies in the case of infections and MDR pathogens.

## 6. Discussion

Given the epidemiological importance of AC and its socio-economic impact, the choice of the best antibiotic therapy is of fundamental importance for the correct management of the patient [12,89]. Physicians should know the principles of clinical and pharmacological treatment for patients affected by AC since in patients with septic shock the biliary origin of peritonitis is a risk factor for mortality at multivariate analysis (OR 3.5; 95% CI 1.09–11.70, *p* = 0.03) [74].

It is clear how source control is the core and definitive treatment for AC. The standard of care in patients with both AC and AAC is early laparoscopic cholecystectomy [18,19,20,21,22]. Source control can be achieved with radiological or endoscopic treatments [38]. In fact, when treated only with antibiotics, patients show recurrence of symptoms in 2.5–22% of cases [23].

Nevertheless, the goal for the treatment of moderate to severe AC is source control; antimicrobial therapy plays a pivotal role in preventing surgical complications and limiting the systemic inflammatory response. Antibiotic therapy has a fundamental role too, especially in patients affected by moderate or severe AC.

Early empirical antimicrobial therapy along with source control of infection is the cornerstone for a successful treatment which, if inadequate, represents an independent predictor factor of mortality [67,68]. The Surgical Infection Society Guidelines recommend a maximum of four days of antibiotic therapy in patients with severe AC undergoing surgery; on the other hand, they recommend against the use of post-operative antibiotics in patients with mild or moderate AC [69].

In the management of critically ill patients with AC, the choice of the antimicrobial regimen may be challenging. It is recommended to start broad-spectrum intravenous antibiotics within the first hour from the onset of septic shock symptoms, and within six hours in other cases [70,71,72]. In critically ill patients, the choice of antimicrobial regimen may be complex, especially for healthcare-associated infections caused by resistant bacteria.

Unfortunately, inconsiderate antibiotic use and increased hospital admissions related to an increase in average age led to a diffusion of antibiotic-resistant strains of bacteria. The resistances vary among nations, regions, and even single institutions. It is therefore necessary to promote, as recommended by recent guidelines, the notion that bile culture or blood culture (in the case of organic dysfunction signs) is necessary.

It is therefore fundamental to identify the microorganism and to choose the correct targeted therapy in order to avoid the selection of multi-resistant strains. Moreover, it is necessary to define the precise antibiotic resistance panel in each hospital [10,75,83].

Correct empirical antibiotic therapy for community-acquired bile infections should be active against Gram-negative bacteria, aerobes, or anaerobes. There is no evidence about the necessity to use specific drugs against Gram-positive germs, except for immunosuppressed or transplanted patients. Complex antibiotic therapies are needed to set up an efficient empirical therapy for healthcare-associated biliary infections [74]. Strong recommendations are available about the duration of therapy: a maximum of four days of antibiotic therapy is recommended after source control in patients with severe AC. This therapy should be modified when microbiological examinations are available (targeted therapy).

## 7. Conclusions

The goal for the treatment of moderate to severe AC is source control, and antimicrobial therapy plays a pivotal role in preventing surgical complications and limiting the systemic inflammatory response. The appropriate use of antibiotic agents is crucial and should be integrated into good clinical practice and standards of care.

## Figures and Tables

**Table 1:** Empiric antibiotic treatment in patients with AC with preserved renal function [8,9,83].

Diagnosis	Common Pathogens	Recommended Treatment *	Daily Dose	Duration of Treatment
Community-acquired AC No risk of MDR organisms Early source control	Enterobacteriaceae Anaerobes Enterococci	Amoxicillin–clavulanic acid	2.2 g three times a day	Single shot or 1–2 days
		Ceftriaxone + metronidazole	2 g per day + 0.5 g three times a day	
		Cefotaxime + metronidazole	2 g three times a day + 0.5 g three times a day	
Community-acquired Older symptoms Immunocompromised/frail patients Late surgery	Enterobacteriaceae Anaerobes Enterococci	Piperacillin–tazobactam	4.5 g three times a day	4–5 days
		Ertapenem	1–2 g per day	

* In the case of beta-lactams allergy: ciprofloxacin 0.4 g twice daily + metronidazole 0.5 g three times a day or moxifloxacin 0.4 g per day. If septic shock: meropenem 2 g three times a day + linezolid 0.6 g twice daily or imipenem–cilastatin 1 g three times a day + linezolid 0.6 g twice daily or tigecycline 0.05 twice daily. When using tigecycline, a loading dose (100 mg) is required and an anti-pseudomonal drug (e.g., piperacillin–tazobactam) must be added. In the case of septic shock, antibiotic therapy must be administered according to the clinical course.

**Table 2:** Antibiotic treatment for AC is suspected or documented for specific resistant pathogens [69,86,88].

Pathogen	Antibiotic
Methicillin-resistant *S. aureus*	Tigecycline ^1^
	Linezolid ^2^
Vancomycin-resistant *E. faecium* or *E. faecalis*	Tigecycline ^1^
	Linezolid ^2^
Extended-spectrum beta-lactamase-forming species (*E. coli*, *Klebsiella* spp.)	Tigecycline ^1^
	Ceftolozane–tazobactam ^3^
	Ceftazidime–avibactam ^3^
	Imipenem–cilastatin
	Meropenem
	Ertapenem
	Fosfomycin (no monotherapy)
*Acinetobacter* spp.	Tigecycline ^1^
	Sulbactam
	Cefiderocol ^3^
Carbapenem-resistant *Enterobacteriaceae* (CRE, non-MBL-producer (e.g., KPC, Oxa-48)	Ceftazidime–avibactam ^3^
	Cefiderocol ^3^
	Meropenem–vaborbactam (KPC)
	Meropenem (high dose)
	Tigecycline ^1^
Carbapenem-resistant *Enterobacteriaceae* (MBL-producer, e.g., NDM, VIM)	Ceftazidime–avibactam ^3^
	Aztreonam
	Cefiderocol ^3^

^1^ If required, combination with antibiotic against *Pseudomonas*; ^2^ if required, combination with antibiotic against Gram-negative and anaerobic spp.; and ^3^ if required, combination with antibiotic against anaerobic spp.
